# “I Believe More in the Ability of the Small Person to Make Big Changes”: Innovation and Social Entrepreneurship to Promote Public Health in Israel

**DOI:** 10.3390/ejihpe13090130

**Published:** 2023-09-13

**Authors:** Keren Dopelt, Nila Mordehay, Shir Goren, Aviya Cohen, Paul Barach

**Affiliations:** 1 Department of Public Health, Ashkelon Academic College, Ashkelon 78211, Israel; nilhahro@edu.aac.ac.il (N.M.); shir11goren@edu.aac.ac.il (S.G.); aviyac11223@edu.aac.ac.il (A.C.); 2 Department of Health Policy and Management, School of Public Health, Faculty of Health Sciences, Ben Gurion University of the Negev, Beer Sheva 84105, Israel; 3 College of Population Health, Thomas Jefferson University, Philadelphia, PA 19107, USA; pbarach@gmail.com; 4 Interdisciplinary Research Institute for Health Law and Science, Sigmund Freud University Vienna, Vienna A-1020, Austria; 5 Department of Surgery, Imperial College School of Medicine, London SW7 2AZ, UK

**Keywords:** social entrepreneurship, social innovation, motivations, barriers, public health, Israel

## Abstract

Social entrepreneurship has grown worldwide in recent decades as it attempts to create and implement innovative solutions to social and environmental issues through business strategies. The aim of this study was to explore what motivates public health social entrepreneurs to act, the challenges and barriers they face, achievements, and competencies required for success. As such, we interviewed 15 social entrepreneurs in Israel. Budget issues, regulatory barriers, and struggles against powerful companies were the frequent barriers to success. The interviewees indicated several achievements at the health policy level by positioning and becoming an authority in the field, positively influencing other people’s lives. They highlighted the importance of creativity, determination and courage, leadership, and the ability to persevere in the face of overwhelming adverse odds as essential for the social entrepreneur’s success. Social entrepreneurship in public health is essential when struggling with health disparities. Nevertheless, recognizing that social entrepreneurship is not a substitute for methodological government planning and accountability is crucial.

## 1. Introduction

Global health inequality is deepening. The social, political, and environmental concerns contributing to inequality range from social tensions, rising unemployment, and weakened representative democracy to severe weather events, corruption, and inadequate infrastructure [1]. The interconnectedness of these concerns makes it difficult to address them using traditional solutions. Social entrepreneurship, defined as a person who explores business opportunities that have a positive impact on their community, is key to success in addressing global inequality and reducing health disparities [2]. Social entrepreneurship has grown worldwide in recent decades as it attempts to create and implement innovative solutions to social and environmental issues through business strategies [3,4]. The persistence of pressing wicked issues—intractable social issues that require new ways of thinking, learning, and doing [5]—is fueling an explosion in social entrepreneurship. 

A few decades ago, “nonprofit entrepreneurship” may have seemed an oxymoron. The need for social entrepreneurship has grown as governments have struggled and failed to address pressing social and environmental challenges effectively, and the willingness of organizations and people to “change the world” has increased [6,7]. Social entrepreneurship is a new arena characterized by blurring disciplinary boundaries, unifying contrasts, and pooling resources [8]. Social entrepreneurs prioritize the needs of marginalized or underserved communities and strive to create a more equitable and just society [9]. Social entrepreneurship is vital in addressing societal and community problems, promoting sustainability, and empowering marginalized groups in society to achieve their full potential [10,11]. Social entrepreneurship can help bridge the gaps between the public and private sectors, applying resources and expertise to address critical public health needs such as poverty, homelessness, and food deserts [12] through effective collaborations with government agencies [13]. The social entrepreneur works for the social benefits of a large group in the population or society as a whole [14]. Contributions to health policy, reducing service gaps, and promoting health are at the core of public health [15]. Roy et al. [16] provide evidence that engagement in social enterprise endeavors has the potential to yield favorable effects on mental well-being, self-sufficiency, self-esteem, health-related behaviors, the attenuation of stigmatization, and the cultivation of social capital. These outcomes collectively contribute to the enhancement of overall health and holistic well-being. Nevertheless, there is little research on the motivations for social entrepreneurship’s underlying factors of individuals’ intention to create a new social enterprise [17,18]. 

Barendsen and Gardner [19] contend that the founder’s background can catalyze the inception of a social enterprise. Asarkaya and Keles-Taysir [20] explored Turkish social entrepreneurs’ life narratives, revealing that their past encounters with specific experiences and individuals significantly influenced their trajectory. Van Ryzin et al. [21] posit that individuals possessing higher levels of education, occupational status, and prior experience in employment and business are more inclined toward adopting the role of social entrepreneurs. Williams and Nadin [22] elucidate that social entrepreneurs present a dual array of motivations: individualistic or societal. Individualistic motives encompass the aspiration for greater autonomy, freedom from hierarchical authority, or pursuing a fresh vocation post-retirement. Societal rationales encompass the desire to aid others or enhance the environment. Koe Hwee Nga and Shamugananthan [23] reveal that social entrepreneurs exhibit kindness, receptiveness to novel ideas, and heightened self-expectations regarding job performance compared to their counterparts. Sastre-Castillo et al. [24] further contribute by indicating that the value most closely associated with an entrepreneurial mindset is openness to embracing change.

The current study aims to fill this gap by exploring the experience and perspectives of social entrepreneurs in public health to help identify strategies and practices for leveraging the power of entrepreneurship to improve population health. We hypothesized that social entrepreneurs are motivated by an inner desire to address unmet public health needs and achieve sustainable impact through innovative approaches and community engagement. 

## 2. Methods

### 2.1. Context—The Public Health System in Israel

Israel’s health system is predominantly public [25]. All residents are entitled to comprehensive public health insurance [26]. The Israeli Ministry of Health is responsible for providing public health services. The public health division includes the units responsible for policymaking and guidelines for regional health departments providing community-based services. The regional leadership is provided by public health physicians, public health nurses, epidemiologists, environmental engineers, nutritionists, health promoters, and other public health-related professionals [27].

### 2.2. Participants

Eighteen public health social entrepreneurs were contacted by e-mail to explain this study’s purpose and ask if they agreed to participate. Fifteen social entrepreneurs consented to participate. 

### 2.3. Data Collection

We conducted a prospective qualitative study using a pretested semi-structured individual interview guide (Appendix A). The topics that guided the interview guide question development were based on the literature and public health social entrepreneurship studies [28]. 

Three senior public health experts from Ashkelon Academic College and Ben Gurion University of the Negev validated the interview guide for clarity, accuracy, and relevance using the content validation method. The interview guide was pilot tested with one social entrepreneur and two senior public health researchers involved in health equality programs to ensure a smooth interview flow and verify comprehension of the questions. We added one question and refined two questions following the pilot phase. The experts agreed that the interview guide corresponds well to the topic and reliably addressed the goals of this study. Information collected during the interviews included the description of the social initiatives, the motivations and barriers, successes and failures, and background demographic details of the interviewee. Interviewees shared their recommendations regarding the need to ease and shorten the bureaucratic obstacles and processes for future social entrepreneurs. 

All interviews were conducted between December 2021 and January 2022 over the Zoom App due to COVID-19 social distancing restrictions. The interviewer was a Public Health undergraduate student (SG), trained in qualitative research methods and supervised by this study’s senior staff, experts in qualitative methods (KD and PB). No relationships were established between the interviewer and this study’s participants before this study’s commencement. Each interview was audio recorded, lasted between 20 and 40 min (average 25.10 ± 8.33), and was transcribed verbatim in Hebrew in a standardized format. The research team discussed the emerging key themes and data richness during the interview, and some of the interviews were analyzed in parallel with the data collection. We derived new hypotheses as a result of this ongoing data analysis.

### 2.4. Data Analysis

Two researchers (KD and PB) analyzed all interviews using a thematic analysis method [29]. The analysis included themes arising from the research topics, data, and review of the public health social entrepreneurship literature. In the first data stage of the analysis, all the interviews were read twice to achieve comprehensive knowledge (saturation) and understanding of the data. Researchers identified categories and themes related to this study’s objectives in the second stage. In the last stage, themes were redefined to include quotes and examples based on the transcripts. The themes and quotes were translated into English at the final step. The translations from Hebrew to English were validated using the retranslation method [30] and a standardized codebook to ensure the validity of the translations from Hebrew to English. We conducted an ongoing internal quality audit using the Consolidated Criteria for Reporting Qualitative Studies (COREQ) checklist [31] to determine whether the data were collected, analyzed, and reported consistently according to the study protocol. 

## 3. Results

### 3.1. Participants

Eleven female and four male public health social entrepreneurs participated in this study, of ages 31–76 years (average 47.33 ± 14.04). All interviewees agreed to reveal the name of their social initiative (Table 1). 

### 3.2. Themes

The data analysis resulted in four main themes that emerged from the interviews:

- Motivations to act (sub-themes: internal motivational factors and external motivational factors),
- Challenges in the establishment and operation of the initiative (sub-themes: budget issues, regulatory challenges, and feeling similar to ‘David against Goliath’),
- Achievements of the initiatives (sub-themes: new procedures and legislation, positioning as an authority in the field, impact on people’s lives and self-empowerment), and
- The virtues of the social entrepreneur.

#### 3.2.1. Theme I: Motivations to Act

All interviewees talked about establishing the initiative, starting from the ideation stage until the initiative began to operate in the field and prosper. Sharing made it possible to identify internal and external motivational factors that motivated them. Nine of the fifteen interviewees identified the importance of internal motivational factors in supporting their social initiative. 

Internal motivational factors

Internal motivational factors are a product of the personal experience of the interviewees, who generally experienced unmet needs, difficulties in getting support, and service implementation gaps (in relationships, knowledge, service, etc.). Nine of fifteen interviewees felt a responsibility and a need to fill these gaps for other citizens coping (or will cope in the future) with similar challenges based on their personal experience. For example, interviewee 14, who struggles with post-traumatic anxiety, initiated the rehabilitation of the mind through movement:

*“Basically, my idea was to teach from my experience that it is both mental coping in all its shades... and also my knowledge of movement that I have developed over the years... to bring it into “Enosh,” the largest mental health association in Israel was a dream for me”*.

Some interviewees talked about a disappointing experience in receiving care and their interactions with the health system when they or their relatives were sick and experienced poor service and inattention from the medical staff. This experience gave them the desire to change how they and others could deal with the health system. Interviewee 13 shared his founder’s story:

*“She got sick and was admitted to the hospital where she worked, and suddenly she became a patient. She said she was lying in bed as if she didn’t exist. The doctors were talking to each other and the nurses about what should be done to her. ‘I’m here! Let them ask me!’ Then She swore that if she could, she would establish an organization that would represent the patients and strengthens them in dealing with the health system”*.

Interviewee 10 described a similar experience:

*“Once, I came to a medical center with sharp pain, and they didn’t believe me. They told me that it was probably imaginary labor. In the end, I arrived alone at the hospital, and it led to a premature birth. Then as I delved deeper into the effects between gender and medicine, I decided to found a body that represents women”*.

Some interviewees felt empowered by their physical activity. They described it as an inner ‘enlightenment’ and wanted to “spread the word” to everyone possible. Interviewee 2 recalled:

*“I played volleyball, and when I returned home, I had two insights: one, only we decide on our priorities in life because we constantly have excuses... the other, suddenly, something I had before the wedding and the children that disappeared came back to me. And suddenly, the opportunity to experience it again enlightened me. I couldn’t leave it to myself because other mothers surely feel exactly like me. Who give the same excuses and therefore I have to act”*.

External motivational factors

The primary motive among six of the interviewees was described as an external motive, either when offered a job or an external trigger that caused them to develop the initiative or join an international ongoing initiative. Interviewees who started establishing the initiative as a response to a job offer understood from the beginning the existing gaps in the health system and the importance of the initiative to advance public health. Interviewee 7 recalled:

*“I was offered to set up a project to prevent smoking, and as soon as I started going deeper, I realized something was interesting about it. I can take my abilities in the field of prevention, and I will use them to prevent smoking”*.

An external trigger influenced interviewee 11, but she also described the deep sense of mission that accompanied her during the process:

*“We founded the association following the polio outbreak that occurred in Israel in 2013, and as part of this outbreak, the Ministry of Health decided that we would have to complete polio vaccines for a group of children who did not receive them... we looked at what was happening in the world, and we realized that such challenges would continue to happen, and we decided to establish Midaat... In my eyes, public health is a significant civic issue, and I believe that health is significant for creating an equal and just society”*.

Two initiatives were imported from abroad: the Healthy Cities Network and La Leche League. Interviewee 5 commented:

*“This is a program of the World Health Organization that started in 1987”*.

#### 3.2.2. Theme II: Challenges in the Establishment and Operation of the Initiative

The interviewees shared the challenges and barriers they faced during the establishment of and running of the initiative.

Budget issues

Six interviewees shared the difficulties of raising funds and limited budgets. Interviewee 13 said:

*“You need money to do things. Today we live on donations. An entrepreneur needs financial resources”*.

Regulatory challenges

Most interviewees talked about the pervasive hyper-regulation and the difficulties in dealing with the various branches of the health system, including the Ministry of Health, hospitals, health funds, and government offices that oversee health and wellness services in Israel. Interviewee 10 addressed this as follows:

*“The health system is a huge body. Some didn’t understand why this woman suddenly came and thought she could tell the doctors what to do. I don’t come from the medical field, so what is my legitimacy to come and demand to be part of the national council and the committees and influence health insurance protocols as a patient? It is not a standard thing.”* Interviewee 5 emphasized: *“The Ministry of Health is interfering. Their vision is very narrow, and our vision is very broad and holistic”*.

Interviewee 12 elaborated on the regulatory difficulties:

*“It was a tremendous difficulty. Everyone I talked with and told that nurses were going to refer patients all laughed and said there was no way it was going to work. We have a very complex regulation here because a nurse can’t just give a patient’s phone number to a student. You must sign a confidentiality agreement. So, you must go through the whole legal aspect of each health fund”*.

Feeling similar to ‘David against Goliath’

The interviews highlighted the difficulties and complexity starting from when the idea was born until its successful implementation. All spoke about the challenges mentioned, but many times there was also a feeling of “weak versus strong” and going up against all odds. For example, interviewee 2 talked about the world of sports, where a dominant male hegemony makes it difficult for women to enter this world:

*“Directors of sports departments, heads of unions and associations, the majority are men. There is complete male control. When I wanted to establish a league of mothers, the mayor kicked me out of his office”*. Interviewee 7 encountered a severe problem as her venture deals with very powerful companies in terms of power, money, and interests:

*“I was told this is a problem that cannot be solved. The tobacco companies have a lot of money. There is no chance to move anything. Full of politics, full of interests. I’m like David against Goliath”*.

#### 3.2.3. Theme III: Success of the Initiatives

The interviewees indicated several achievements at the health policy level by positioning and becoming an authority in the field, positively influencing other people’s lives. At a personal level, this contributed to their growth and empowerment.

New procedures and legislation

New procedures and legislation are significant achievements that affect the health of the public and result from hard work and lobbying the decision-makers. Everything must be backed up by studies and statistics. Legislation is the fruit of tireless efforts by many and is considered one of the most important principles in health promotion. New legislation achievements can change reality and benefit many people, and interviewees said this was the most significant change they could achieve. For example, interviewee 7 proudly shared:

*“One achievement is the law banning advertising, and electronic cigarettes were included. And second, we handled the comparison of the tax on rolling cigarettes to regular cigarettes. The initiative got appreciation from the World Health Organization for these two achievements”*.

Positioning as an authority in the field

The interviewees were proud that their initiative developed from a nascent idea to a recognized professional authority. Interviewee 8 shared:

*“We are members of some committees in the Knesset, the welfare committee for the children’s rights, the Ministry of Health contacts us during the breastfeeding week. It says everything”*.

Interviewee 2 spoke about international resonance:

*“I am one of the only women’s sports entrepreneurs in the world who managed to break male hegemony, which is now being replicated in many countries in the world”*.

Interviewee 4 also shared their achievement:

*“Even the Ministries of Health and Education consult with us. We have become the country’s largest center of knowledge for treating vaccine resistance in all aspects. And that is the big thing”*.

Impact on people’s lives

Social entrepreneurs strive to impact people’s lives positively and to create equality, fairness, and social justice in society. Interviewee 12 talked about the importance of helping elderly citizens who struggle with chronic diseases:

*“There were exciting and amazing stories about how we actually saved people. We realized that this was a tremendous result...”*

Interviewee 14 elaborated on the help needed for the mentally challenged while changing the perceptions on healing and providing inspiration for new projects in the mental health field:

*“This is something new, groundbreaking that brings healing, it brings relief to suffering... It brings solutions in places that have not been touched and inspires many other projects. In general, bringing a healing approach to mental health is the point. Bringing a healing approach, not holding the contenders to a level of minimal functioning that they are simply some kind of burden or won’t make too much noise, but to see that it is possible to build the person’s coping journey into a journey of development and recovery”*.

Interviewee 11 spoke about an indirect effect on people:

*“In the end, tens of thousands of people we talked to and hundreds of thousands of people who received the content we produced and were helped by them directly and indirectly and millions of people who heard us in the media that we were able to influence as part of our activities. I am happy that I could do something that has a positive impact. It is not without frustrations because we never get to do everything we want, and our influence is always limited. But every time I meet someone who says thank you for receiving information that helps him, for helping him to make a decision”*.

Self-empowerment

One of the initiatives’ secondary goals was to support entrepreneurs’ empowerment. Although this is not an achievement, it came up in all the interviews. All the entrepreneurs experienced frustration and disappointment and faced many difficulties and obstacles along the way. They did not give up and had complete convictions in their idea and the righteousness of their ways. The challenges strengthened their resolve on a personal level. 

For example, interviewee 4 sees the initiative as the most significant thing they have done:

*“That is bigger than anything else I’ve done in my life. I was an officer in the army, learned skydiving, founded kindergartens, was an educator, and many things. And ‘Mehusgan’ orders of magnitude are bigger than anything I’ve done in my life. It’s not money, it’s not profit, just a tendency to do good. It’s fun. All the people who connect with ‘Mehusgan’ are great. Amazing doctors. Everyone. Wonderful people are All around us, and we are in an amazing place. It drains us of many, many, many hours at the expense of the family and everything, but every time we think we have reached a peak, it reaches a new peak.”* Interviewee 6 added: *“I learned a lot about myself and many things beyond what I expected. I had to learn how to build a website. I had to call hospitals and collect donations. I don’t know if it changed me, but it gave me the strength to do other things”*.

Interviewee 2 explained:

*“It made me a stronger mother, a more aware mother, and the successes and the fact that other mothers made me realize that I can influence others. That is very, very strengthening and empowers me and my ability. It made me very sensitive to people and attentive. My motto as the director of the largest women’s league organization in Israel is that there are many good people like me, and we give them the platform to grow and grow”*.

Interviewee 10 summed up nicely:

*“My involvement in the association has greatly changed me. I believe more in the ability of the small person to make a big change”*.

#### 3.2.4. Theme IV: The Virtues of the Social Entrepreneur

The participants discussed the essential qualities of an effective social entrepreneur. The majority agreed that creativity and innovation were crucial, as well as tolerance and perseverance to face and overcome challenges without giving up. Interviewee 14 emphasized:

*“The entrepreneur needs to be creative, think outside the box, and be innovative. He also needs patience and a willingness to work hard. There is a possibility that the dream will not come true. Now the idea is not to give up on the dream and not to be afraid of difficulties, to be dedicated and go to the end even if it takes time”*.

Interviewee 2 added:

*“In sports, you live by rules, and to do entrepreneurship in sports, you must break this thing. Because you can’t enter and fit into any cube that was there before”*.

Interviewee 1 mentioned the need to deal with situations of uncertainty:

*“You have to be a person who sees a challenge and sets out to conquer it. Someone willing to take risks, who knows how to deal with situations of uncertainty”*.

Interviewee 12 emphasized the more practical level, the need to identify gaps and ensuring is needed versus what is available:

*“To create an initiative that will be successful, you have to do a lot of testing and distilling and understanding which is the right answer to a need that exists and is significant, it needs to be examined very comprehensively and to understand who the different players are, who the competitors are, what exists and what does not exist”*.

Interviewee 13 spoke about flexibility and the ability to convince and build diverse coalitions:

*“Many social initiatives are founded thanks to crazy people. As an entrepreneur, you need to be flexible with learning ability, persuasiveness, and know how you create coalitions because alone, in many cases, it is difficult for you to do”*.

Interviewee 12 averred:

*“One of the dangers of entrepreneurs is falling in love with an idea because what happens when you fall in love with an idea? You become blind, and you don’t look at other options. The process requires tremendous flexibility, listening to what is happening, and rapid changes and corrections. When I obscure or am busy with my worldview, I can miss and even sabotage the initiative”*.

## 4. Discussion

Social entrepreneurship has gained widespread importance in recent years as a powerful way to address pervasive societal gaps [32,33]. Our findings highlight the motivations, challenges, and achievements of public health entrepreneurs in Israel and the key character traits required for effective social entrepreneurship in the field of public health. The findings provide a comprehensive understanding of social entrepreneurs’ experiences, perspectives and identify strategies and best practices for leveraging social entrepreneurship to address public health challenges. 

Social entrepreneurs are often driven by a desire to make the world a better place and to create sustainable solutions to pressing issues [17]. The driving factors for many social entrepreneurs were a keen sense of fairness and deep motivation, an internal process that occurred within the individual, that brought with it the intention to act and the persistence to persevere [19,20,34]. Motivation influences entrepreneurial behavior in three ways: the choice of the individual, the intensity of the action, and the persistence of the action [35]. The theory of self-direction [36] distinguishes between types of motivation according to the reasons or goals that cause action. The most basic distinction is between internal motivation, which relates to doing something because it relates to an inner experience, while external motivation relates to doing something because it gives rise to a particular distinct result.

The health system in Israel is changing at an unprecedented rate, and entrepreneurship is one way to deal with these challenges. In developed countries such as Israel, social entrepreneurship is on the rise due to the decline and shortcomings of the welfare state. In less developed countries, social entrepreneurship arises from a combination of government mistrust, indifference in the private sector, and the inability of the government to provide effective services to people [37]. Being an entrepreneur means taking responsibility and leading actions to change it. This reality can be a personal journey for the entrepreneur himself or a social fact [38]. A central theme in our interviews dealt with the motivation for the initiative. The personal exposure of the social entrepreneur to health service shortfalls and the needs not met by the existing policy often triggered a change or renewal of policy to address the real-world gaps.

Our findings highlight the importance of internal and external motivational factors. Internal motivational factors are personal experiences: a desire to improve an existing situation, direct experience with inequality, opacity on the part of the system and the medical staff, dealing with bureaucracy, and lack of knowledge. The external motivational factors in this study included being offered a job or taking part in an international initiative. Once they launched the initiative, the entrepreneurs immediately understood its importance and the potential to contribute and cause lasting change. The interviewees internalized the reasons for action and assimilated them so that the activities performed out of external motivation were ultimately done out of increased self-directedness [39].

The interviewees shared the difficulties they faced and reinforced the idea that their motivation came from an internal place and a desire to improve people’s health in their dealings with their health and social systems. The motives of these entrepreneurs were noble and often not self-interested. They identified a local concern and brought creative ideas that could affect a recognized social problem [40]. This study and previous studies, e.g., the studies in [40,41] found that internal motivational factors are stronger than external ones in initiating and sustaining action.

The second theme dealt with the challenges encountered by entrepreneurs during the development of their initiative. We found that facing challenges or obstacles in the early stages of establishing new ventures is normal and can be a powerful driving and positive process [17]. Studies have identified that entrepreneurs face several challenges, such as regulation, lack of financial assistance, information, and excessive taxation, and the need to face them with an open mind and mental flexibility [17,42,43]. 

In contrast, the entrepreneurs in our study were exposed to the daily needs and difficulties, as well as the problems and shortcomings of Israeli policies in health and public health. In the interviews, several barriers came up that the entrepreneurs had to deal with, such as the lack of organizational management knowledge, recruiting partners, budgets, experience dealing with government officials and recognition from the establishment, heavy-handed regulation, and a lack of understanding in the field in which the venture is engaged [44,45].

Social entrepreneurship needs ongoing financial support and dedicated budgets to achieve its goals, especially in the early stages of establishing and operating the venture. The interviewees emphasized that access to funding was the main difficulty. On the other hand, the health system presents numerous regulatory obstacles to entrepreneurs, and the entrepreneur must overcome complicated technical processes, which significantly slow down the pace of progress. Financial and regulatory problems were a common theme among all interviewees. Some entrepreneurs felt similar to “David against Goliath” at the beginning of their journey. They felt “small” and powerless, struggling against powerful and wealthy multinational companies and capricious regulators.

Another theme dealt with the achievements and products of the initiative. Becoming a social entrepreneur has a profound impact—people choosing social entrepreneurship to do good for others was repeated in all the interviews, and it deeply impacted the social entrepreneurs [46]. Social entrepreneurs succeed in gathering legitimacy for their actions and support for their efforts in the face of many obstacles and despite uncertainty. They increase their resources and the scope of their activities through volunteers, public support, donations, and funding from public sources [47]. The interviewees noted their main achievements were at the policy level, positioning the initiative as a trusted authority, lobbying for the passing of legislation, raising knowledge and awareness, and positively impacting other people’s lives. While the purpose of a business organization is to generate profit for shareholders, the purpose of a social organization is to create social impact, and its success is measured by its social impact measures [48].

The research findings suggest that the entrepreneurial experience can cause personal empowerment. This phenomenon is complex and includes emotional and rational elements [49]. Entrepreneurs usually approach entrepreneurship from a personal, deeply emotional, and responsible point of view. The entrepreneurial journey becomes a very personal journey in which the values, beliefs, assumptions, positions, and personal strengths of the entrepreneur are leveraged and regularly tested simultaneously. The life experience of the entrepreneur is significant and leads to personal growth, increased awareness, and self-confidence [50]. When entrepreneurs go through the entrepreneurial experience, they seek to find meaning and personal fulfillment and enable a change in the way they are perceived by others, perhaps to improve their self-image, confidence, and personal power [51]. Many of the interviewees said that they chose this arduous journey to fulfill something deeper within themselves, find their place in the world by helping others, and create deeper meaning in their lives while creating changes in the world around them [52].

The last topic dealt with the virtues of the social entrepreneur. The interviewees mentioned a cluster of characteristics, such as creativity, determination and courage, patience, persuasiveness, leadership, and immunity to criticism and failure, needed to overcome challenges and persevere. In addition, we noted that the entrepreneur must show mental flexibility and not be “in love with their own idea”. These findings are consistent with previous studies that showed that successful social entrepreneurs are committed to the social mission and work to create significant change and social value [53], identify a gap in needs [54], define a vision [55], succeed in overcoming obstacles and challenges [53] (Short et al., 2009), and succeed in mobilizing the needed resources to advance the initiative [56].

### Limitations

Our study has several limitations. First, we had to select the interviewees from a small number of public health initiatives and had limited participants. Fortunately, all the participants we approached agreed to be interviewed. Second, the study results reflect the unique experiences of an Israeli public health entrepreneur. Israel is recognized internationally as the “startup nation (https://sifted.eu/articles/israel-startup-ecosystem-tech-vc-brnd, accessed on 17 March 2023)”, which may not be generalizable to other countries within diverse legislations, cultural and social contexts. Third, the interviews were transcribed from Hebrew, the native language of Israel. This may have increased the chances for variations in the interpretation of our data. We made many efforts to ensure methodological rigor and validity of the translations from Hebrew to English by using a standardized codebook, meeting frequently, sharing and comparing our results, and performing a pilot analysis. Throughout this study, we conducted an ongoing internal quality audit during our meetings, adapted from Tong et al. [31], to determine whether the data were collected, analyzed, and reported consistently according to the study protocol.

## 5. Conclusions

This study considerably advances the understanding of the drives and motivations of public health social entrepreneurs. The themes of resource mobilization, financial viability, cross-disciplinary collaboration, and systems strengthening were woven throughout the interviews. At the micro level, the interviewees talked about their motivations to make a social change, the difficulties, and the assertive personality characteristic required for social entrepreneurship success. At the meso level, they shared the feeling of struggling against powerful and rich companies when they had only limited financial means and influence but with a strong faith in the righteousness of their way to do good for the public and reduce health disparities. It is nicely reflected in the ‘David against Goliath’ metaphor. At the macro level, the regulatory challenges that the state piles up can be overwhelming, and the ability to stand firm and lead change to the point of creating new guidelines or legislation is critical. In addition to motivation, entrepreneurs must consider the personal and professional challenges and time demands involved in effective social entrepreneurship in public health. Social entrepreneurs initiate reforms and structural changes in the social sector, where social value is created by introducing new forms of activity with the aim of solutions to be sustainable for political change.

### Implications of This Study

While social entrepreneurship can help address critical public health needs, it is essential to recognize that it is not a substitute for government planning and accountability. Government services and funding remain crucial for ensuring a strategic health equality vision, access to healthcare, disease prevention, and health education for all members of society. Social entrepreneurship in public health should be seen as a complement to government services, not a replacement. Future studies should compare social entrepreneurs in public health versus social entrepreneurs in other fields (such as education and welfare) and examine whether there are meaningful differences. More research is needed to understand the implications for capacity building, financing, scaling, and policy making. Finally, more must be done to evaluate the contributions of public health entrepreneurship in promoting population health.

## Figures and Tables

**Table 1:** Social entrepreneur initiatives list.

No	Initiatives Translated into English (Hebrew)	Initiative Goal	Characteristics
1	Shades (Gvanim)	Establish a therapeutic and educational center for parents and children	Female, 51, Married + 3, MBA
2	Netball for moms (Mama-net)	Form a Mothers’ Netball League	Female, 43, Married + 2, BA
3	Walking circles	Establish walking groups	Female, 73, Married + 3, MA
4	Vaccinated kindergarten (Mehusgan)	Make reliable medical information about vaccinations and the prevention of infectious diseases accessible to the community, kindergartens, parents, and kindergarten teachers	Male, 42, Married + 3, BEd
5	Healthy Cities	Start an association of local authorities, government ministries, health funds, and academia working together to develop health and sustainability at the local level	Female, 76, Married + 3, MD, MPH
6	Sheets from home	Provide new and fun bedding for children with cancer	Female, 45, Married + 3, BA
7	The project to eradicate smoking	Prevent smoking initiation among teenagers and young adults	Female, 48, Married + 2, MA
8	La Leche League	Breastfeeding encouragement	Female, 40, Married + 4
9	Yoga in the neighborhood	Make the practice of yoga accessible to the entire population	Female, 31, Married, BA
10	A healthy foundation for women (Keren Bri’a)	Improve health services, accessibility, and care for women	Female, 39, Married, MA
11	Aware (Mida’at)	Advance health promotion in the fields of medical treatment and preventive medicine	Female, 40, Married, MA
12	Embrace (Hibuk)	Enhance access to health services in the community for chronically ill patients and alleviating loneliness for those who, due to their medical condition, rarely leave their house	Male, 37, BA
13	The Association of Patients’ Rights	Provide assistance and guidance in dealing with various medical conditions, focusing on the realization of the rights in the healthcare system in Israel	Male, 69, Married, MHA
14	Mind and Fitness	Improve the lifestyle for those dealing with mental disabilities	Male, 42, Single, BA
15	Mortal (Enosh)	Provide rehabilitation and treatment for people facing a mental disability and their families, and promote policies in the field	Female, 45, Married + 3, MA

## Data Availability

The data presented in this study are available on request from the corresponding author.

## Appendix A. Social Entrepreneur’s Interview Guide

1. Can you tell me about yourself: age, marital status, profession, workplace, etc.?
2. Tell me about the social initiative in which you are involved, what is the purpose of the initiative, when did the initiative begin, how did you think about it, and what needs you did you try to meet.
3. Where did you get the motivation to implement the initiative? How did you manage to progress the initiative from a dream to reality?
4. What obstacles did you encounter along the way? Can you give an example? Have you had moments of despair where you thought of giving up? What helped you to go on?
5. What are your most significant achievements concerning the initiative as far as you are concerned? What are your failures?
6. Would you like to add anything else?
